# Spectroscopic in-depths of upconverting NaZr_2_(PO_4_)_3_ phosphors for LIR-based thermometry

**DOI:** 10.1039/d3ra02126b

**Published:** 2023-07-12

**Authors:** Lakshmi Mukhopadhyay, Sasank Pattnaik, Vineet Kumar Rai

**Affiliations:** a Laser and Spectroscopy Laboratory, Department of Physics, Indian Institute of Technology (Indian School of Mines) Dhanbad 826004 Jharkhand India vineetkrrai@iitism.ac.in vineetkrrai@yahoo.co.in +91-326-223 5404/528; b DRDO, Terminal Ballistics Research Laboratory Chandigarh-160030 India

## Abstract

This study employed solid-state synthesis to develop the green emitting Er^3+^-Yb^3+^:NaZr_2_(PO_4_)_3_, NASICON material. Using Rietveld refinement, the crystallographic variables of the synthesized phosphors were precisely calculated. The upconverting phenomenon was seen with naked eye when exposed to 980 nm laser radiation. The intermediate excited state dependency on the unusual photon number dependence on the green and red emission has been understood using the steady-state rate law equations. Further, the temperature sensing performances with good repeatability were compared with different laser densities, and it was found that the prepared phosphors could be an ideal material for upconverting and temperature sensing applications.

## Abstract 2

This study employed that the green-emitting Er^3+^-Yb^3+^:NaZr_2_(PO_4_)_3_, NASICON phosphors are very much promising in optical thermometry applications.

## Graphical abstract

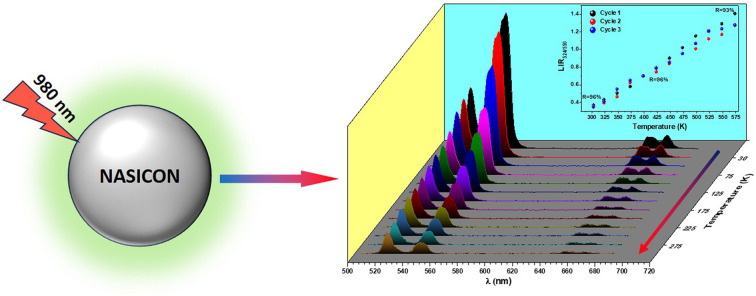

## 1. Introduction

The search for suitable energy materials for fuel cells and rechargeable batteries has gained attention in recent years because of the limitation of non-conventional sources and rapid decrement of inexpensive oil. Lithium-ion batteries show potential application as an energy storage system in portable and wearable electronic devices.^1–3^ Lithium-ion batteries face several shortcomings, including high cost, limited energy densities, the low abundance of lithium, and low efficient recycling.^3–5^ Again, in lithium batteries, liquid electrolytes, such as LiClO_4_ dissolved in propylene carbonate, are used, which impart several disadvantages, such as device failure due to electrode corrosion, limited operating temperature range, and unsuitable shapes.^6^ Thus, it is important to select a solid electrolyte in order to avoid the above-mentioned problems. Currently, research is focused on sodium-ion rechargeable batteries, which can operate at room temperature, owing to their low cost, the natural abundance of sodium, and wide geographical distributions.^3–5^ The sodium superionic conductor (NASICON) compounds with the structure Na[Ge, Ti, Zr]_2_(PO_4_)_3_ are studied by researchers since the early 60s owing to their unique structure for sodium-ion batteries. NASICON materials possess several interesting features, such as high ionic conductivity at room temperature or elevated temperature, high thermal stability, low thermal expansion coefficient, resistance to radiation damage, low solubility in water, large surface area, and capability to accommodate ions. These properties of NASICON compounds suggest their suitability in various industrial applications, such as in gas sensors, fuel cells, rechargeable batteries, and nuclear waste immobilization.^7,8^ NaZr_2_(PO_4_)_3_ is an interesting NASICON-type anode material that consists of PO_4_ tetrahedra sharing a corner with ZrO_6_ octahedra and linked to the three-dimensionally interstitial space where the alkali and alkaline-earth metal ions can partially occupy. The open and strongly bonded structure makes possible the fast movement *i.e.*, the high mobility of the Na^+^ ions tunnelling through polyhedra chain (O_3_ZrO_3_(Na)ZrO_3_O_3_).^9–11^ In order to synthesize thermally stable efficient luminescent materials, the incorporation of the f-block element in the NASICON structure has been carried out by several researchers. The downconversion luminescence study based on the lanthanide doped (Eu^2+^, Sm^3+^) NASICON materials has been reported for a broad range of applications, including white light emitting diodes (WLEDs).^10,12^ The upconversion (UC) emission characteristics of the NASICON materials can also be studied by choosing suitable dopant ions.^13,14^ Er^3+^ ions are widely used as dopant ions owing to their large number of ladder-like energy levels, high emission lifetime, and two thermally coupled levels (TCLs), which result in intense green emission. Although Er^3+^ ions have a direct energy level corresponding to the 980 nm excitation, Yb^3+^ ions are used as sensitizers owing to their broad absorption cross-section compared to that of Er^3+^ ions.^15,16^ The temperature sensing study is very popular in recent days to monitor the most important physical parameter ‘Temperature’. The accurate temperature detection of inaccessible objects and intracellular cells is very difficult with the contact thermometry method. The non-contact temperature measurement is very essential in the field of coal mines, oil refiners, medical diagnosis, metallurgical, food manipulation, and testing.^16,17^ In non-contact thermometry, the temperature is measured depending on optical parameters such as luminescence intensity ratio (LIR), luminescence lifetime, emission bandwidth, and peak wavelength. The LIR technique is the most used technique among researchers as it is insensitive to the external environment such as pressure, and light source, whereas other optical techniques strongly depend on the atmosphere.^17,18^ The energy difference between levels ^2^H_11/2_ and ^4^S_3/2_ of Er^3+^ ions is ∼780 cm^−1^, which lies in TCL's energy gap range. The LIR of the ^2^H_11/2_ → ^4^I_15/2_ and ^4^S_3/2_ → ^4^I_15/2_ levels thus can be used for the optical thermometry study.

In this work, the upconversion (UC) luminescence and temperature sensing behaviour using the luminescence intensity ratio technique of Er^3+^-Yb^3+^:NaZr_2_(PO_4_)_3_ phosphors were investigated under 980 nm laser radiation. The structural, optical, and electrochemical properties of the generated phosphors were also investigated.

## 2. Experimental details

### 2.1. Synthesis of materials

Er^3+^-Yb^3+^:NaZr_2_(PO_4_)_3_ NASICON material has been prepared by the solid-state reaction technique. The starting reagents to prepare the host material are sodium carbonate (Na_2_CO_3_), zirconium dioxide (ZrO_2_), and sodium dihydrogen phosphate (NH_4_H_2_PO_4_), which follows the reaction given as,

1 Na_2_CO_3_ + 4ZrO_2_ + 6NH_4_H_2_PO_4_ = 2NaZr_2_(PO_4_)_3_ + CO_2_ + 6NH_3_ + 9H_2_O

For doping Er^3+^ and Yb^3+^ ions into the host material, erbium oxide (Er_2_O_3_) and ytterbium oxide (Yb_2_O_3_) were taken. Er^3+^ ions were doped by varying different concentrations from 1 mol% to 9 mol% and after optimizing Er^3+^ ions, Yb^3+^ ions were incorporated by varying the concentration from 1 mol% to 15 mol%. The weighed starting materials were taken in an agate mortar and ground for 2 h with the help of acetone and kept in a muffle furnace at 400 °C for 1 h and again annealed at 1200 °C for 4 h.

### 2.2. Characterization techniques

X-ray diffraction (XRD) patterns of the prepared NASICON materials were obtained using a Bruker DA advance X-ray diffractometer equipped with Cu-K_α_ radiation (*λ* = 1.5406 Å). The surface morphology of the prepared phosphors was studied by field emission scanning electron microscopy (FESEM) Supra 55 with an air lock chamber. The Raman spectra of the synthesised phosphors were recorded at room temperature by using a Renishaw Raman spectrometer fitted with an argon ion laser at 514.5 nm. The FT-IR spectra of the prepared phosphors were obtained using a Perkin-Elmer spectrum-2 spectrophotometer. The UV-Vis-NIR double-beam spectrophotometer from Agilent's Cary-5000 series was used to record the diffuse reflectance spectra. The UC emission spectra of all developed phosphors were recorded on a Princeton Acton SP 2300 triple turret grating monochromator with a photomultiplier tube. The samples were placed within a tiny handmade heater that was controlled using a varivolt and monitored by a k-type thermocouple to conduct the temperature dependence study.

## 3. Results and discussion

### 3.1. Structural and morphological analysis

**Fig. 1:**
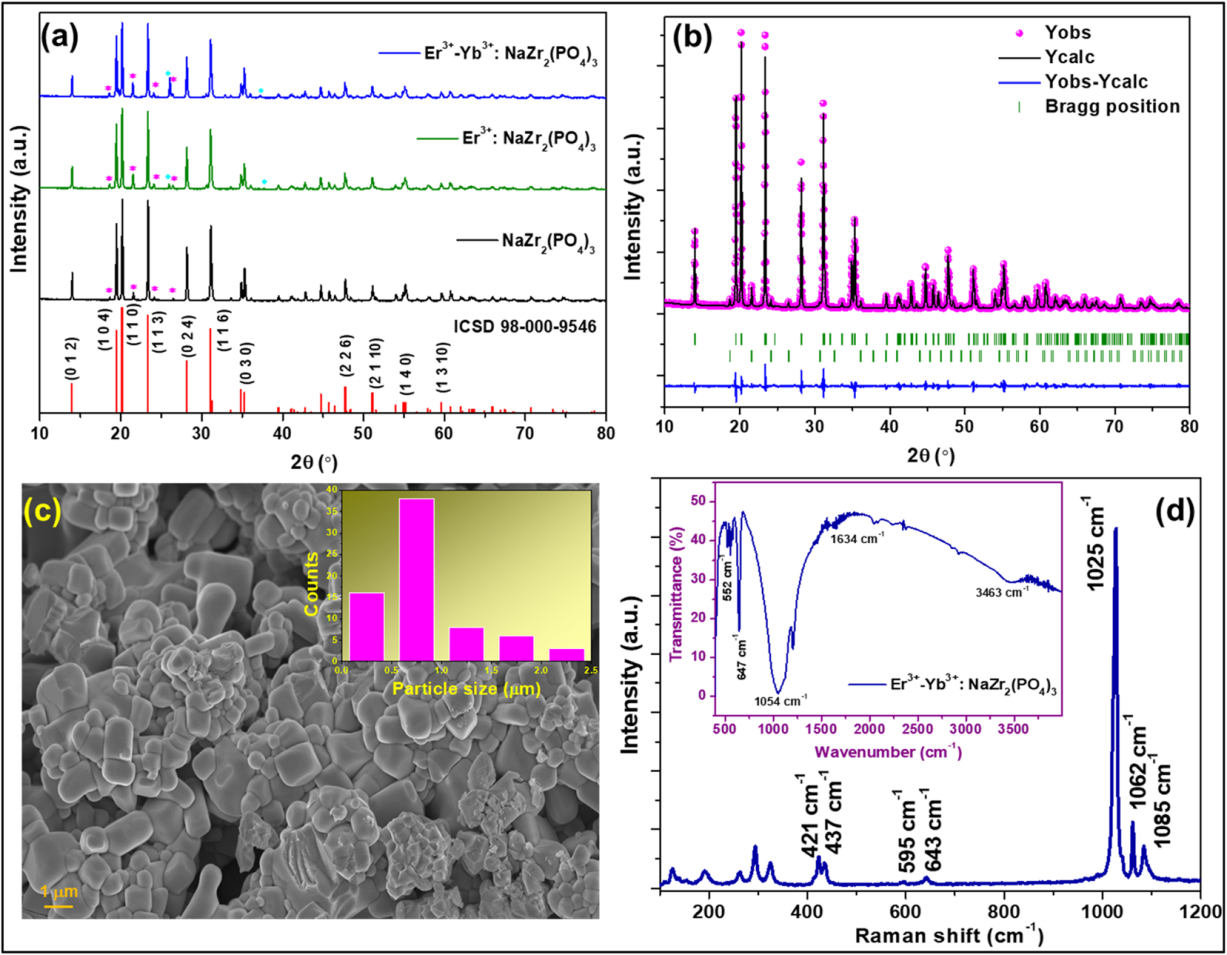
X-ray diffraction patterns of (a) NaZr_2_(PO_4_)_3_, 7 mol% Er^3+^:NaZr_2_(PO_4_)_3_, and 7 mol% Er^3+^-10 mol% Yb^3+^:NaZr_2_(PO_4_)_3_ phosphors, (b) Rietveld refinement pattern of 7 mol% Er^3+^-10 mol% Yb^3+^:NaZr_2_(PO_4_)_3_ phosphors, (c) FESEM image of 7 mol% Er^3+^-10 mol% Yb^3+^:NaZr_2_(PO_4_)_3_ phosphors, (d) Raman spectrum (inset: FT-IR spectrum) of the 7 mol% Er^3+^-10 mol% Yb^3+^:NaZr_2_(PO_4_)_3_ phosphors.

The XRD pattern was recorded to confirm the formation of the crystalline phase of the Er^3+^-Yb^3+^:NaZr_2_(PO_4_)_3_ phosphors in the range of 10–80° at room temperature {Fig. 1(a)}. In the diffraction pattern, the Bragg peaks well matched with those from the ICSD pattern 98-000-9546, which has a hexagonal structure with space group *R*3̄*c* and space group #167. The maximum intensity peak in the XRD pattern was observed at ∼20.2° corresponding to the (1 1 0) Bragg plane, whereas the other main diffraction peaks were seen at ∼14.02°, ∼19.5°, ∼23.4°, ∼28.2°, ∼31.1°, ∼35.3°, ∼47.8°, and ∼55.2° refers to the (0 1 2), (1 0 4), (1 1 3), (0 2 4), (1 1 6), (0 3 0), (2 2 6), and (1 4 0) diffraction planes, respectively. Some small impurity peaks have been observed in the diffraction pattern, which correspond to zirconium diphosphate (ZrP_2_O_7_) and erbium phosphate (ErPO_4_) denoted in the figures as ‘*’ and ‘♦’, matching the standard ICSD patterns 98-002-4854 and 98-016-7090, respectively having cubic (*Pa*3̄) and tetragonal (*I*41/*amd*) phases. The impurity peaks appear in very small intensities, thus, they may not have any significant impact on the structural, optical, and conducting properties.^19^ The ionic radii of Na^+^, Zr^4+^, P^5+^, Er^3+^, and Yb^3+^ ions are 102 pm, 72 pm, 38 pm, 89 pm, and 86.8 pm, respectively. To validate the substitution of doped ions in the possible ion site present in the host materials, the acceptable ionic radii percentage difference (*D*_r_) was calculated using the formula given as,^19,20^2
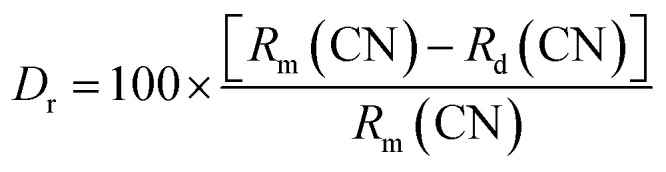
where, *R*_m_(CN) and *R*_d_(CN) represent the ionic radii of the host cation and the doped ion, respectively. The calculated values of *D*_r_ are 12.7% and 14.9% (less than 30%) for Er^3+^ and Yb^3+^ ions, respectively, to be doped in the Na^+^ ion site suggesting successful doping of dopant ions. Further, to establish the phase purity of the prepared phosphors, Rietveld refinement of the XRD patterns of the undoped and doped NaZr_2_(PO_4_)_3_ phosphors using FullProf software was carried out. Fig. 1(b) depicts the refinement profile of Er^3+^-Yb^3+^:NaZr_2_(PO_4_)_3_ phosphors and the refined parameters with the quantification of the impurity phases of the prepared phosphors are summarized in Table 1.

**Table 1:** Quantitative Rietveld refinement parameters of NaZr_2_(PO_4_)_3_ phosphors

Samples	Phase	*R*_p_	*R*_wp_	*R*_exp_	*χ* ^2^	Cell parameters (Å)	Cell volume (Å^3^)	Quantification
NaZr_2_(PO_4_)	Hexagonal	11.3	11.3	5.78	3.73	*a* = 8.80018(6), *b* = 8.80018(6), *c* = 22.7601(3)	1526.47(2)	96.2(2)%
	Cubic					*a* = 8.2439(3), *b* = 8.2439(3), *c* = 8.2439(3)	560.28(4)	3.78(7)%
Er^3+^:NaZr_2_(PO_4_)_3_	Hexagonal	11.4	11.2	6.25	3.20	*a* = 8.80849(8), *b* = 8.80849(8), *c* = 22.7793(3)	1530.64(3)	91.9(2)%
	Cubic					*a* = 8.2615(2), *b* = 8.2615(2), *c* = 8.2615(2)	563.88(3)	7.15(8)%
	Tetragonal					*a* = 6.8620(6), *b* = 6.8620(6), *c* = 6.012(1)	283.09(8)	0.90(3)%
Er^3+^-Yb^3+^:NaZr_2_(PO_4_)_3_	Hexagonal	11.8	12.2	6.11	3.98	*a* = 8.8094(1), *b* = 8.8094(1), *c* = 22.7772(4)	1530.85(3)	88.6(8)%
	Cubic					*a* = 8.2744(3), *b* = 8.2744(3), *c* = 8.2744(3)	566.51(3)	5.9(5)%
	Tetragonal					*a* = 6.8369(2), *b* = 6.8369(2), *c* = 5.9873(5)	279.87(2)	5.4(1)%

In order to examine the surface morphology of Er^3+^-Yb^3+^:NaZr_2_(PO_4_)_3_ phosphors FESEM analysis was carried out (Fig. 1(c)). The images showed that the prepared phosphors were agglomerated and the particles had different sizes. From the Gaussian distribution depicted in Fig. 1(c) inset, it has been estimated that the size of most of the particles was in the 500–600 nm range.

### 3.2. Raman and FTIR analysis

To investigate the structures of the molecules present in the prepared phosphors, vibrational spectroscopic tools such as Raman and Fourier transform infrared (FTIR) spectroscopy were utilized. The Raman spectrum of the prepared NASICON materials was recorded in the range of 100–1200 cm^−1^ as shown in Fig. 1(d). The spectra consisted of several bands in the range between 320–1089 cm^−1^, which are ascribed to the PO_4_^3−^ internal vibration whereas the bands arising in the range 100–300 cm^−1^ are referred to the external modes such as lattice phonon modes, and the translational vibrations and liberations (pseudo-rotations) of the polyhedral.^20,21^ The most intense Raman band was observed at ∼1025 cm^−1^, which can be assigned to symmetric stretching (*ν*_1_) vibration, and the bands at ∼1062 cm^−1^ and ∼1085 cm^−1^ are due to the asymmetric stretching (*ν*_3_) vibration of PO_4_^3−^. The other low intense Raman bands arising at ∼595 cm^−1^ and ∼643 cm^−1^ are corresponding to the asymmetric bending (*ν*_4_) and that at ∼421 cm^−1^ and ∼437 cm^−1^ are due to the symmetric bending (*ν*_2_) vibration of PO_4_^3−^.

The FTIR spectrum of the Er^3+^-Yb^3+^:NaZr_2_(PO_4_)_3_ phosphor was recorded to record the vibrational bonds from in material {inset: Fig. 1(d)}. The broad absorption band peaking at ∼1054 cm^−1^ is due to asymmetric stretching (*ν*_3_) of P–O tetrahedra. The peaks in the range 520–650 cm^−1^ arise due to the asymmetric bending mode (*ν*_4_) and below this symmetric bending mode (*ν*_2_) of the PO_4_^3−^ group is responsible. The vibrational bands at ∼1634 cm^−1^ and 3463 cm^−1^ are due to the O–H bond present in the material.^21^ The intensity of these impurity bands appears very low in the FTIR spectrum, which represents the better emission intensity of the present phosphor material.

### 3.3. Spectroscopic investigation

**Fig. 2:**
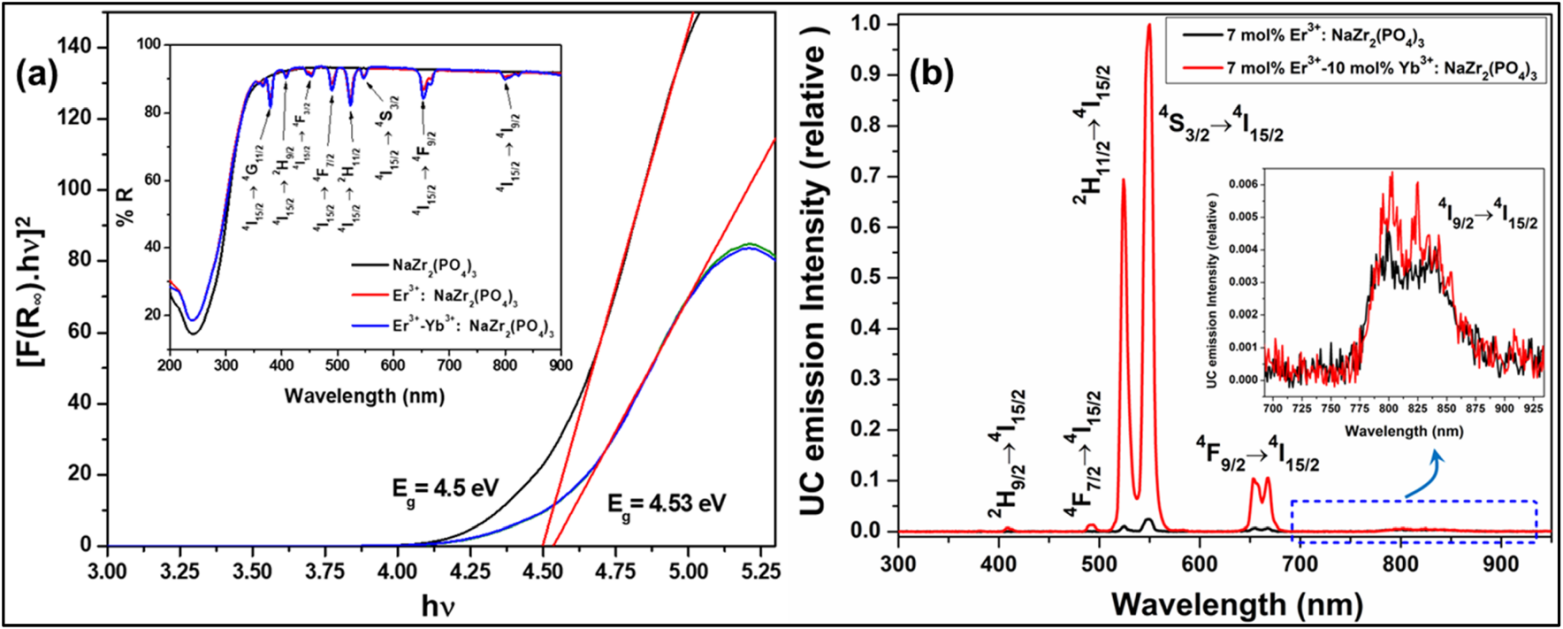
(a) Tauc plot of Er^3+^/Er^3+^-Yb^3+^:NaZr_2_(PO_4_)_3_ phosphors, (inset: diffuse reflectance spectra of 7 mol% Er^3+^ and 7 mol% Er^3+^-10 mol% Yb^3+^:NaZr_2_(PO_4_)_3_ phosphors. (b) UC emission spectra of 7 mol% Er^3+^-10 mol% Yb^3+^:NaZr_2_(PO_4_)_3_ phosphors and the inset shows the enlarged spectra of (804, 824) nm peak.

Diffuse reflectance spectra for all synthesized phosphors at room temperature were monitored in the UV-vis-NIR range to study the absorption characteristics of the materials. Fig. 2(a) shows the DRS data for both undoped and co-doped NaZr_2_(PO_4_)_3_ phosphors in the range from 200 to 900 nm, with a step size of 0.5 nm. The BaSO_4_ powder was adopted as a reference for baseline correction. While the host lattice has been attributed to the broad peak that appears between 200 and 350 nm, the sharp band that appears after this wavelength is caused by f–f transitions from rare-earth ions. The transitions responsible for the peaks at 380, 408, 454, 490, 524, 546, 654, and 799 nm are ^4^I_15/2_ → ^4^G_11/2_, ^4^I_15/2_ → ^2^H_9/2_, ^4^I_15/2_ → ^4^F_3/2_, ^4^I_15/2_ → ^4^F_7/2_, ^4^I_15/2_ → ^2^H_11/2_, ^4^I_15/2_ → ^4^S_3/2_, ^4^I_15/2_ → ^4^F_9/2_, ^4^I_15/2_ → ^4^I_9/2_, respectively.^22^ The DRS spectra, in addition to recognising the peaks, assist in determining the optical bandgap of the synthesized phosphors. The Kubelka–Munk equation was employed to analyse the DRS spectra in order to evaluate the optical bandgap of the phosphor:^23^3
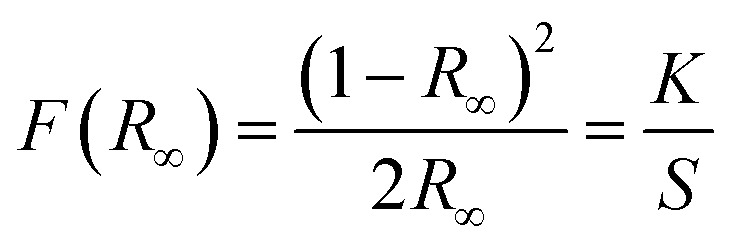

4 [*F*(*R*_∞_)*hυ*]^2^ = *C*(*hυ* − *E*_g_)^*n*^

where *K* and *S* stand for the absorption and scattering coefficients, respectively, and *hv* is the input photon energy; *F*(*R*_∞_) is the Kubelka–Munk function; *R* is the diffuse reflectance of the phosphor; The *n* value determines the type of optical transition, with *n* = 1 denoting direct allowed transitions, *n* = 2 denoting non-metallic materials, *n* = 3 denoting directly prohibited transitions, *n* = 4 denoting indirect allowed transitions, and *n* = 6 denoting indirect forbidden transitions.^24^*E*_g_ in eV represents the optical band gap. Here, *n* = 1 produced the best match. The optical bandgaps for NaZr_2_(PO_4_)_3_, Er^3+^:NaZr_2_(PO_4_)_3_, and Er^3+^-Yb^3+^:NaZr_2_(PO_4_)_3_ phosphors were calculated to be 4.5 eV, 4.53 eV, and 4.53 eV, respectively. The minute change in the optical bandgap due to the incorporation of Er^3+^ and Yb^3+^ ions could be a result due to the Moss–Burstein effect.

The frequency UC emission spectra of the optimized Er^3+^/Er^3+^-Yb^3+^:NaZr_2_(PO_4_)_3_, NASICON phosphors recorded in the range 450–850 nm are displayed in Fig. 2(b). The UC spectrum of Er^3+^:NaZr_2_(PO_4_)_3_ phosphors exhibit prominent green and red emission bands peaking at ∼524 nm, ∼550 nm, and ∼(654, 668) nm corresponding to the ^2^H_11/2_ → ^4^I_15/2_, ^4^S_3/2_ → ^4^I_15/2_, ^4^F_9/2_ → ^4^I_15/2_ and ^4^F_7/2_ → ^4^I_15/2_ transitions of Er^3+^ ions, respectively. Also, a weak blue band at ∼490 nm and a NIR band in the range 800–830 nm have been noticed [inset of Fig. 2(b)] corresponding to the ^4^F_7/2_ → ^4^I_15/2_ and ^4^I_9/2_ → ^4^I_15/2_ transitions of Er^3+^ ions, respectively. The codoping of Yb^3+^ ions in the Er^3+^:NaZr_2_(PO_4_)_3_ phosphors results in intensity enhancement of all the emission bands. In the optimized 7 mol% Er^3+^-10 mol% Yb^3+^:NaZr_2_(PO_4_)_3_ phosphors the green, red, and blue emission band intensities increase ∼87, ∼28, and ∼2 times, respectively, in comparison to the optimized 7 mol% Er^3+^:NaZr_2_(PO_4_)_3_ phosphors. The significant enhancement in the Er^3+^-Yb^3+^ codoped phosphors is due to the energy transfer from Yb^3+^ ions to Er^3+^ ions, which refers to the broad absorption cross-section of Yb^3+^ ions. It can be noted that blue emission intensity does not notably enhance as in the case of green and red emission bands. Thus, the possibility of cooperative energy transfer is ruled out. However, beyond the optimized concentration of dopant ions, the UC emission intensity decreases due to the concentration quenching effect. The concentration quenching phenomenon of the dopant ions occurs because of the non-radiative energy transfer or the cross-relaxation processes between the two neighbouring dopant ions. With the increase of the dopant ion concentrations, the distance between two adjacent dopant ions decreases, which in turn increases the possibility of non-radiative energy transfer. Thus, the energy transfer mechanism occurring between the dopant ions can be analyzed by calculating the critical distance (*R*_c_) between two same adjacent dopant ions with the help of Blasse's equation given as,^25^5
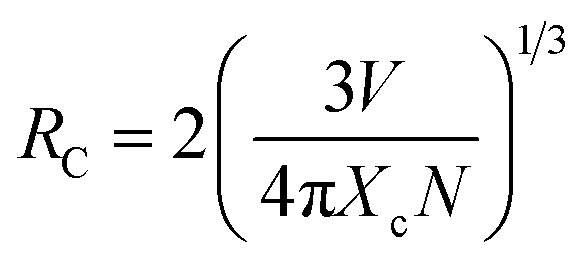
where ‘*V*’ is the unit cell volume, ‘*X*_c_’ refers to the critical concentration of dopant ions, and ‘*N*’ is the number of available crystallographic sites occupied by the dopant ions in the unit cell (*N* = 6). In the case of optimized Er^3+^ and Yb^3+^ phosphors, the values of *R*_c_ were calculated to be ∼19.09 Å and ∼14.21 Å, respectively, which are larger than 5 Å. According to Van-Uitert's law, the exchange interaction is not accountable for the energy transfer in the present case rather the *R*_c_ value suggests multipolar interactions between the dopant ions is responsible for the energy transfer process.

Furthermore, when the laser pump power density of the 980 nm laser excitation increases, it has been observed that the UC emission spectra of Er^3+^-Yb^3+^:NaZr_2_(PO_4_)_3_ phosphors show a higher increment rate in the ∼524 nm band compared to the ∼550 nm as the pump power increases, which in turn increases the luminescence intensity ratio (LIR) of these two thermally coupled levels (TCLs) of Er^3+^ ions. The LIR enhancement with the variation of pump power suggests the temperature generation in the phosphor material, which indicates the suitability of the present phosphor in temperature-sensing applications.

Initially, a 980 nm photon is absorbed by an Er^3+^ ion and promoted to the ^4^I_11/2_ level from the ground state ^4^I_15/2_*via* the ground state absorption (GSA) process. The excited ions at the ^4^I_11/2_ level relax non-radiatively to the ^4^I_13/2_ level, and through the process of excited state absorption (ESA), the ions in the ^4^I_11/2_ and ^4^I_13/2_ states are further excited to the ^4^F_7/2_ and ^4^F_9/2_ states, respectively. The emission at 494 nm can be caused by the radiative transition between ^4^F_7/2_ and ^4^I_15/2_. Also, the ^4^F_9/2_ state was employed to populate the ^2^H_9/2_ state by an ESA process, yielding a peak at 409 nm. The multiphonon relaxation from the ^4^F_7/2_ state populates the thermally coupled levels, *i.e.*, ^2^H_11/2_ and ^4^S_3/2_, which then radiatively relax to the ground state. As a result, photons in the green region are observed at 524 nm and 550 nm, associated with the ^2^H_11/2_ → ^4^I_15/2_ and ^4^S_3/2_ → ^4^I_15/2_ transitions, respectively. The peak in the red region at 654 and 668 nm can be attributed to the Stark sublevels of the ^4^F_9/2_ → ^4^I_15/2_ transitions. A small peak in the NIR range of about 804 nm and 824 nm is responsible for the Stark splitting of the ^4^I_9/2_ → ^4^I_15/2_ transition, as shown in the inset of Fig. 2(b). In the case of Er^3+^-Yb^3+^ co-doped phosphors, UC emission was observed by the ET process in addition to GSA and ESA. Because of the GSA and ET from the Yb^3+^ ions, the population of excited Er^3+^ ions in the ^4^I_11/2_ state for the Er^3+^-Yb^3+^ co-doped system is dominant over the Er^3+^ doped systems. The energy transfer processes that populate the ^4^I_11/2_, ^4^F_9/2_, ^4^F_7/2_, and ^2^H_9/2_ states are given as follows.ET_1_: ^2^F_5/2_ (Yb^3+^) + ^4^I_15/2_ (Er^3+^) →^2^F_7/2_ (Yb^3+^) + ^4^I_11/2_ (Er^3+^)ET_2_: ^2^F_5/2_ (Yb^3+^) + ^4^I_11/2_ (Er^3+^) →^2^F_7/2_ (Yb^3+^) + ^4^F_7/2_ (Er^3+^)ET_3_: ^2^F_5/2_ (Yb^3+^) + ^4^I_13/2_ (Er^3+^) →^2^F_7/2_ (Yb^3+^) + ^4^F_9/2_ (Er^3+^)ET_4_: ^2^F_5/2_ (Yb^3+^) + ^4^F_9/2_ (Er^3+^) →^2^F_7/2_ (Yb^3+^) + ^2^H_9/2_ (Er^3+^)

Moreover, using the steady-state rate equations, the depopulation rates of different metastable states with different pump photon densities could be estimated theoretically as,6
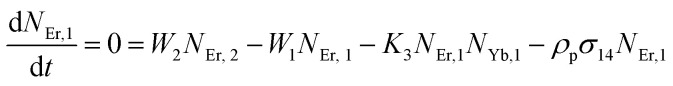
7
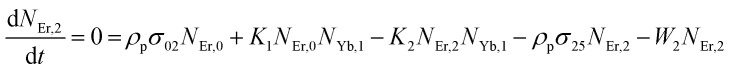
8
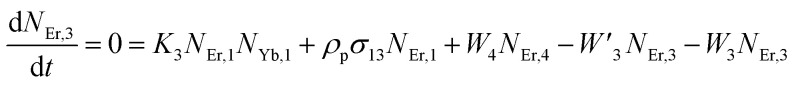
9
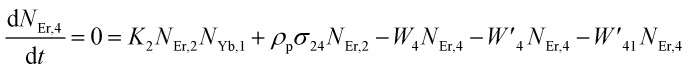
10
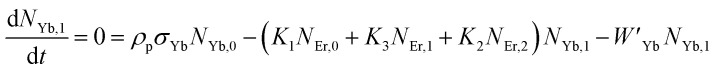
The population densities for Er^3+^ ions at the ^4^I_15/2_, ^4^I_13/2_, ^4^I_11/2_, ^4^F_9/2_ and (^4^S_3/2_, ^2^H_11/2_) levels, are denoted by *N*_Er,k_ (*k* = 0, 1, 2, 3, 4) whereas *N*_Yb,0_ and *N*_Yb,1_ represent the population densities for Yb^3+^ ions at the ^2^F_7/2_ and ^2^F_5/2_ levels, respectively. *K*_1_, *K*_2_, and *K*_3_ indicate the corresponding energy transfer rates of ET-1, ET-2, and ET-3. *W*_1_, *W*_2_, *W*_3_ and *W*_4_ represents the non-radiative decay rates of the ^4^I_13/2_, ^4^I_11/2_, ^4^F_9/2_ and (^2^H_11/2_, ^4^S_3/2_) levels, respectively. *W*′_3_, *W*′_4_ and *W*′_41_ defines radiative decay rates of the ^4^F_9/2_, ^4^S_3/2_, and ^2^H_11/2_ levels, respectively. Whereas, the radiative decay rate of ^2^F_5/2_ of the Yb^3+^ ion is represented by *W*′_Yb_. Whereas *σ*_Yb_ stands for the absorption cross-section between Yb^3+^ ions at levels ^2^F_7/2_ and ^2^F_5/2_, *σ*_*ij*_ stands for the absorption cross-section between Er^3+^ ions at the *i*th and *j*th levels. *ρ*_p_ denotes the pump power density at 980 nm. Since the absorption cross-section of Er^3+^ ions is lower than that of Yb^3+^ ions, it may be believed that processes such as GSA and ESA had a minimal role in filling the metastable states and can thus be neglected.

The non-radiative linear decays from the ^4^I_11/2_ and ^4^I_13/2_ levels dominate ET-2 and ET-3 from Yb^3+^ ions to Er^3+^ ions at low pump power densities. The energy transfer rates from eqn (6) and (7) can thus be ignored. Thus,11
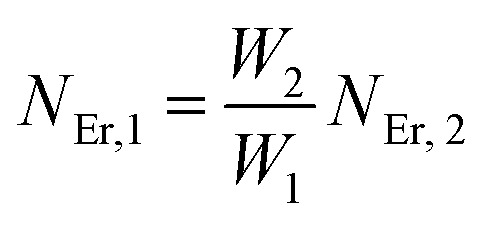
12
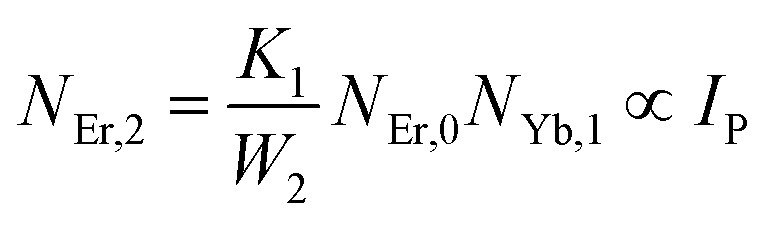
and eqn (10) can be rewritten as,13
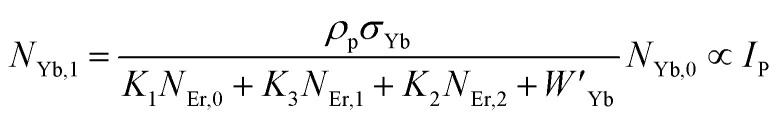


Eqn (11) and (12) may be used to modify eqn (8) and (9), giving the result14
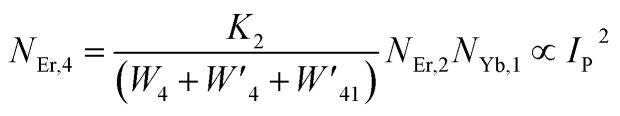
15
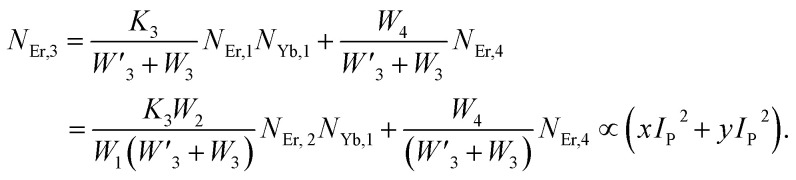


The populations associated with the green and red emissions are represented by eqn (14) and (15), respectively. As can be seen, the populations at each level are quadratic, meaning that two photons are needed to fill levels ^2^H_11/2_, ^4^S_3/2_, and ^4^F_9/2_. It could be assumed that the ET processes must eliminate the non-radiative losses at the ^4^I_11/2_ and ^4^I_13/2_ levels as the pump power density steadily increases. Thus, by leaving out the variables −*W*_1_*N*_Er,1_ and −*W*_2_*N*_Er,2_, eqn (6) and (7) may be simplified as follows:16
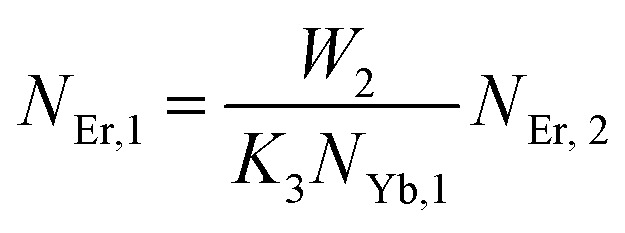
17
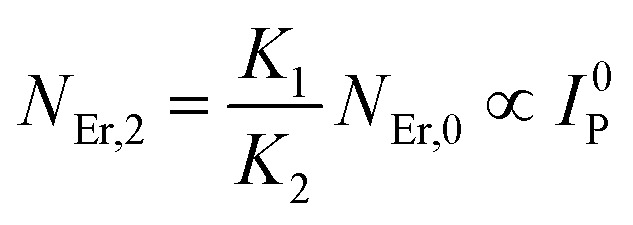
18
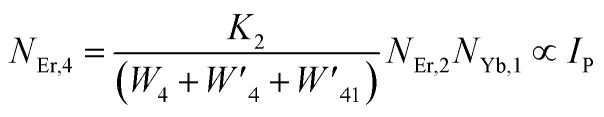
19
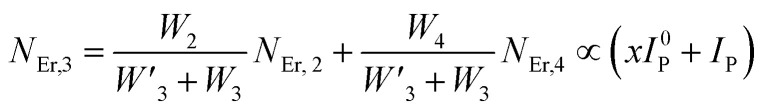


From eqn (18), it can be seen that the population associated with the green emitting level is proportional to a single-photon absorption. Eqn (19), whose first term is independent of the pump photon and second term suggests a single-photon absorption, reflecting the red emission at the same time. According to the above observation, ET-2 becomes efficient at higher pump power densities, non-radiative processes grow quickly, and finally the saturation effect results.

**Fig. 3:**
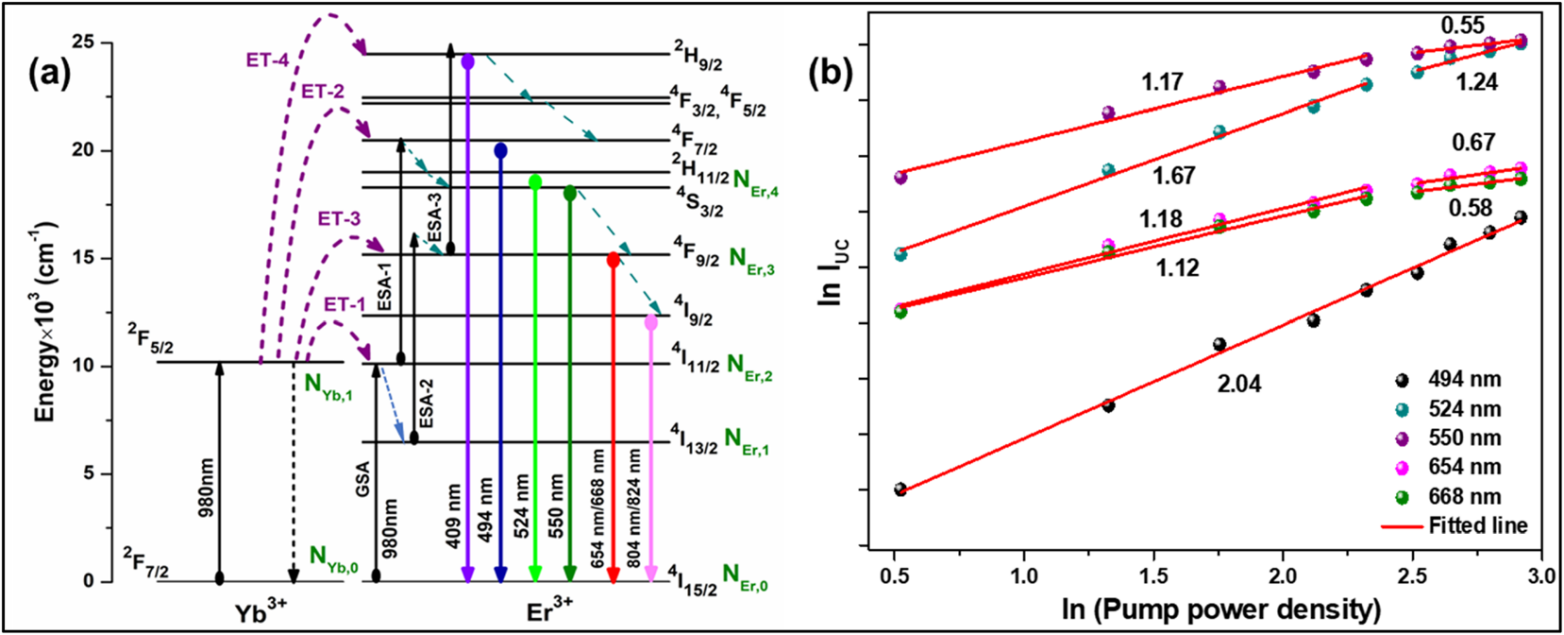
(a) Energy level diagram of Er^3+^-Yb^3+^ codoped system, (b) ln–ln plot of UC emission intensity and laser pump power density.

To verify the above theoretical assumptions, the UC emission was measured utilising a range of laser pump power density ranges (980 nm). According to previous studies, the relationship between the input pump power density (*p*) and the UC emission intensity (*I*_UC_) is non-linear, with I_UC_ ∝ *I*_P_n.^26^ The number of infrared (IR) pump photons necessary to reach specific levels in order to produce UC emission depends on the value of “*n*” in this equation. Fig. 3(b) displays the double logarithmic plot between *I*_UC_ and *P*_pump_, with “*n*” calculated from the slope values. The calculated photon number for the 494 nm, 524 nm, 550 nm, 654 nm, and ∼668 nm are found to be 2.04, 1.67, 1.17, 1.18, and 1.12, respectively, which confirmed the involvement of the two photons for the green and red UC emission bands. However, the slope values tended to decrease at higher pump power densities. The results obtained from the double logarithmic plot suggest that for the green emission, the slope value is nearer to 2, but, for the red emission, it approaches 1. This could be understood by comparing the depletion mechanism of the intermediate state responsible for the UC luminescence. According to Pollnau *et al.*, when the linear decay of the intermediate state is the dominant depletion mechanism, *I*_UC_ ∝ *I*_P_2, whereas, *I*_UC_ ∝ *I*_P_1 when the upconversion (*e.g.*, ESA and ET) becomes the primary depletion mechanism.^27^ The intermediate states for the green and red emissions in the Er^3+^-doped phosphors are ^4^I_11/2_ and ^4^I_13/2_, respectively. Upconversion emerges as the primary depletion mechanism of ^4^I_13/2_ because the linear decay rate of the ^4^I_11/2_ level is relatively higher than that of the ^4^I_13/2_ level. As a result, the green emission has a larger slope value than the red emission. Due to the large concentration of Er^3+^ and Yb^3+^ ions in Er^3+^-Yb^3+^:NaZr_2_(PO_4_)_3_ phosphors, the ET rates between the dopants would increase the efficiency of upconversion from the intermediate state to the emitting state, hence elevating the significance of upconversion. As a result, the slope of the UCL shifts from quadratic to linear.^28^

### 3.4. Temperature sensing behaviour

The temperature-dependent UC emission spectra of Er^3+^-Yb^3+^:NaZr_2_(PO_4_)_3_ phosphors were recorded in the temperature range 303–573 K under 980 nm excitation with fixed laser pump power. To study the LIR variation based on the green TCLs of Er^3+^ ions with temperature variation the emission spectra have been recorded in the wavelength range 510–570 nm. The overall green emission intensity decreases with the increase in temperature without any change in the band position, which is due to an increase in non-radiative relaxation rate and electron–phonon coupling within the sample. It is noticeable that with the increase of temperature, the rate of decrement in UC emission intensity corresponding to the ^2^H_11/2_ → ^4^I_15/2_ transition is higher than that for the ^4^S_3/2_ → ^4^I_15/2_ transition, which results in the LIR increment of these TCLs. The LIR variation with temperature can be described by the Boltzmann distribution, which follows the equation,^29^20
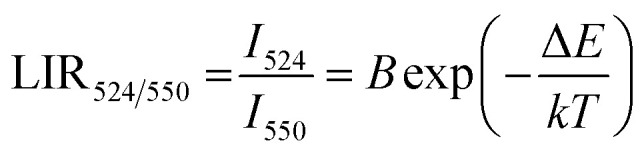
where, *I*_524_ and *I*_550_ represents the integrated intensities of the ^2^H_11/2_ → ^4^I_15/2_ and ^4^S_3/2_ → ^4^I_15/2_ transitions, respectively, *B* is the proportionality constant, Δ*E* is the energy separation between two thermally coupled levels, *k* and *T* are the Boltzmann's constant and absolute temperature, respectively. To examine the temperature sensing performance of the prepared phosphors, first, the pump power density was fixed to 5.78 W cm^−2^, and the LIR change was monitored with the elevation temperature. For Er^3+^-Yb^3+^:NaZr_2_(PO_4_)_3_ phosphors, the LIR varies from 0.43 to 1.47 as the temperature varies from 303 K to 573 K.

**Fig. 4:**
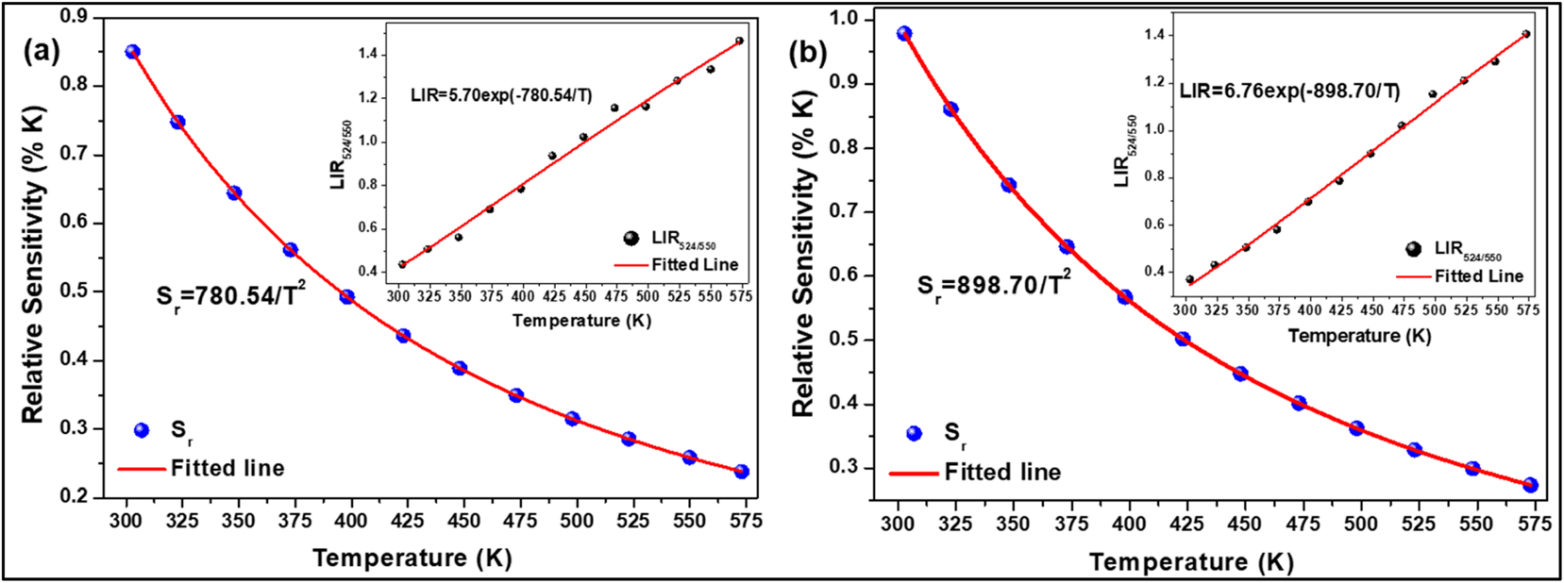
Relative sensitivity (LIR_524/550_ variation) as a function of temperature at (a) 3.76 W cm^−2^, (b) 5.78 W cm^−2^ of 7 mol% Er^3+^-10 mol% Yb^3+^:NaZr_2_(PO_4_)_3_ phosphors.

By fitting the experimental data according to eqn (20), we obtained the temperature sensing parameter, Δ*E*/*k* = 780.54{Fig. 4(a)}. The values of ‘*B*’ and ‘Δ*E*’ were found to be 5.70 and 542.48 cm^−1^, respectively, for the Er^3+^-Yb^3+^:NaZr_2_(PO_4_)_3_ phosphors. However, the impact of laser power density may induce a heating effect which hinders the temperature sensing performance. To further verify the effect of laser-induced heating, we have monitored the temperature sensing performance at a lower pump power density, *i.e.*, at 3.76 W cm^−2^. As can be seen from Fig. 4(b), the temperature sensing parameter enhances to 898.70 with the change in LIR from 0.37 to 1.41 on varying the temperature from 303 to 573 K. Moreover, to quantify the temperature sensing performance of Er^3+^-Yb^3+^:NaZr_2_(PO_4_)_3_ phosphors, the sensitivity was calculated. The relative sensor sensitivity (*S*_r_) is a crucial parameter to validate the phosphor material as a temperature-sensing probe. The relative sensor sensitivity (*S*_r_) can be defined as,^30^21
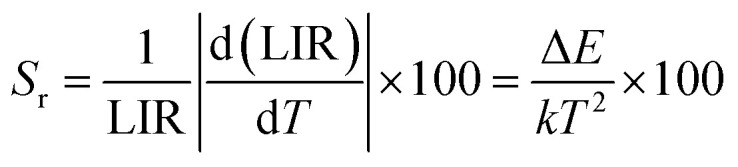
where all terms have their usual meanings. The maximum value of *S*_r_ for Er^3+^-Yb^3+^:NaZr_2_(PO_4_)_3_ phosphors was obtained as 0.98% K^−1^ at 303 K for 3.76 W cm^−2^ of pump power density. In Table 2, we have compared the temperature sensing sensitivity of the present work with other reported works and we have found the Er^3+^-Yb^3+^:NaZr_2_(PO_4_)_3_ phosphors could be utilized for temperature sensing applications.

**Table 2:** Summary of the ^2^H_11/2_, ^4^S_3/2_ → ^4^I_15/2_ TCLs-based temperature sensing performance of different Er^3+^ and Er^3+^-Yb^3+^ codoped materials excited under 980 nm laser

Doped phosphors	Temperature range (K)	Maximum *S*_r_ (% K^−1^)	Ref.
Er^3+^-Yb^3+^:NaYF_4_	223–403	0.36% K^−1^ at 300 K	33
Er^3+^-Yb^3+^:Gd_2_O_3_ phosphor	300–900	0.39% K^−1^ at 300 K	34
Er^3+^:SrSnO_3_	294–372	0.997% K^−1^ at 294 K	35
Er^3+^-Yb^3+^:Na_2_GdMg_2_(VO_4_)_3_ phosphors	303–573	0.976% K^−1^ at 303 K	36
Er^3+^-Yb^3+^:NaZr_2_(PO_4_)_3_ phosphors	303–573	0.98% K^−1^ at 303 K	Present work
Er^3+^-Yb^3+^:Ba_3_La(PO_4_)_3_	298–498	1.17% K^−1^ at 298 K	37
Er^3+^-Yb^3+^:NaBiF_4_ nanoparticle	303–483	1.24% K^−1^ at 303 K	38

**Fig. 5:**
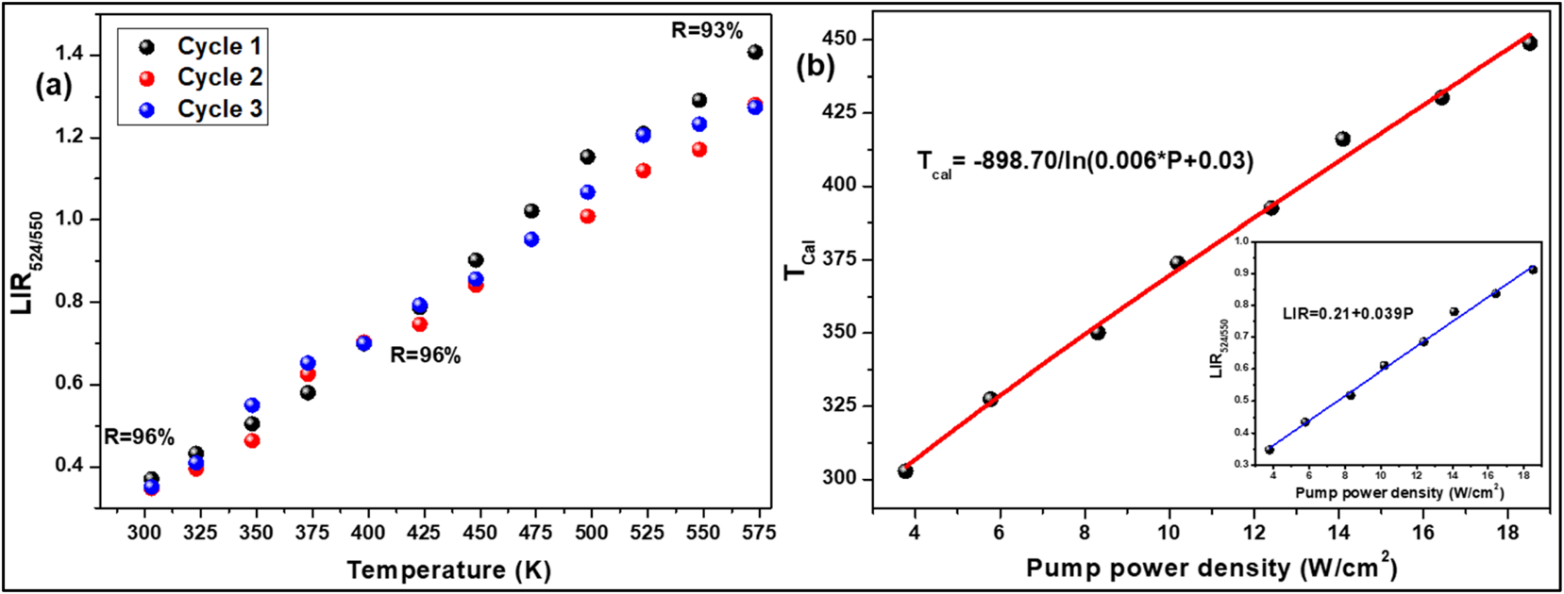
(a) Repeatability of LIR from 300 K to 573 K for 3 cycles, (b) a plot of calculated temperatures *vs.* pump power densities (inset: LIR *vs.* pump power densities) for 7 mol% Er^3+^-10 mol% Yb^3+^:NaZr_2_(PO_4_)_3_ phosphors.

To verify the reproducibility of the experiment, we monitored the LIR change for 3 different cycles in the temperature range from 303–573 K and computed the repeatability (*R*) as,^31^22
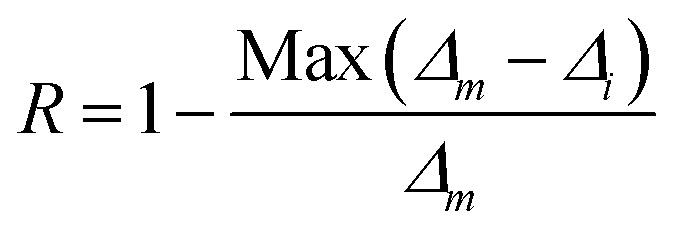
Here *Δ*_*m*_ states the mean LIR value for 3 cycles and *Δ*_*i*_ states the LIR value at temperature *T*. After calculating the *R*-value, we obtained that it was ∼96% at 303 and 423 K and 93% at 573 K {Fig. 5(a)}. The Er^3+^-Yb^3+^:NaZr_2_(PO_4_)_3_ phosphors not only have good sensitivity at low pump power density, but they could also convert some of the absorbed NIR excitations to heat at higher pump power. The inset of Fig. 5(b) shows that the LIR grows linearly with the pump power (*P*), resulting in the equation,^32^

23 LIR = 0.039*P* + 0.21

By comparing eqn (23) to eqn (20), the temperature was determined as24
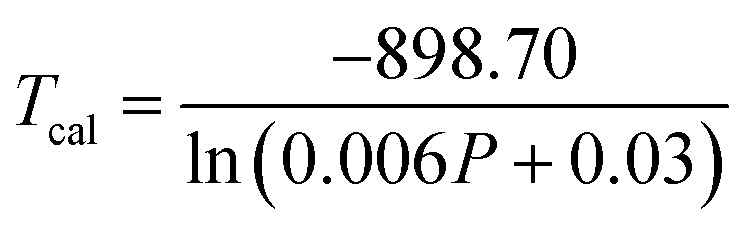


According to the above equation, each pump photon has a unique temperature associated with it that is produced by non-radiative relaxation from the higher energy levels (Fig. 5(b)). According to earlier research, applications such as photothermal treatment require temperatures between 315 and 318 K to kill cancer cells.^32^ Hence, it can be claimed that the criteria may be satisfied by varying the laser pump power between 4.81 and 4.96 W cm^−2^.

## 4. Conclusion

In conclusion, a series of NaZr_2_(PO_4_)_3_ UC green phosphors doped with Er^3+^ and Yb^3+^ ions were synthesised by a solid-state-reaction approach. The Rietveld refinement of XRD profiles offered structural insight into the phases present in the phosphors, and the acquired crystal structure parameters demonstrate that the predicted model is the best fit for the refinement. Raman spectroscopy was used to validate various lattice vibrations. The prepared phosphors have a non-uniform shape, which may be seen in the microstructure. The co-doping of Yb^3+^ ions considerably enhanced the UC green emission, which corresponded to the ^2^H_11/2_, ^4^S_3/2_ → ^4^I_15/2_ transition of Er^3+^ ions, and about 87-fold enhancement was observed at 10 mol% Yb^3+^ doping concentration. The energy-level diagram for UC was examined in depth using steady-state rate law equations. In the temperature dependence UC study, the maximum *S*_r_ values for the optimised phosphor were determined to be 0.98% K^−1^ at 303 K for 3.76 W cm^−2^ of pump power density using the LIR method. As a result of the enhanced UC luminescence and temperature sensing capability, the produced phosphor is a promising contender in the area of optical thermometry.

## Conflicts of interest

There is no conflict of interest.

## Supplementary Material
